# The Relationship between Alcohol Hangover Severity, Sleep and Cognitive Performance; a Naturalistic Study

**DOI:** 10.3390/jcm10235691

**Published:** 2021-12-03

**Authors:** Elizabeth Ayre, Andrew Scholey, David White, Grant J. Devilly, Jordy Kaufman, Joris C. Verster, Corey Allen, Sarah Benson

**Affiliations:** 1 Centre for Human Psychopharmacology, Swinburne University, Melbourne, VIC 3122, Australia; eayre@swin.edu.au (E.A.); andrew@scholeylab.com (A.S.); dawhite@swin.edu.au (D.W.); j.c.verster@uu.nl (J.C.V.); 2 Nutrition Dietetics and Food, School of Clinical Sciences, Monash University, Melbourne, VIC 3800, Australia; 3 Swinburne Neuroimaging, Swinburne University, Melbourne, VIC 3122, Australia; 4 School of Applied Psychology, Griffith University, Brisbane, QLD 4122, Australia; grant@devilly.org; 5 Griffith Criminology Institute, Griffith University, Brisbane, QLD 4122, Australia; 6 Swinburne BabyLab, Swinburne University, Melbourne, VIC 3122, Australia; jkaufman@swin.edu.au; 7 Division of Pharmacology, Utrecht University, 3584 CG Utrecht, The Netherlands; 8 Queensland Police Service Academy, Brisbane, QLD 4108, Australia; allencorey1966@hotmail.com

**Keywords:** alcohol hangover, sleep quantity, sleep quality, executive functioning, working memory, attention

## Abstract

Alcohol hangover (AH) has been associated with poor sleep due to the negative effects of alcohol intoxication on sleep quantity and sleep quality. The aim of the current study was to further explore the relationship between AH severity and sleep using a naturalistic study design. A further aim was to determine whether quantitative aspects of sleep were a mediating influence on the relationship between AH severity and cognitive performance. As part of the naturalistic study design, 99 drinkers were recruited following a night of drinking in an Australian state capital, with breath alcohol concentration (BrAC) measured as participants were leaving the entertainment district. The following morning at home, participants answered online questions regarding their drinking behaviour on the previous evening, current AH symptoms and sleep quality. Participants also completed an online version of the Trail-Making Test B (TMT-B) to assess cognitive performance. The findings reveal the duration of nightly awakenings to be negatively related to six individual AH symptoms as well as overall AH severity. The number of nightly awakenings, sleep quality and total sleep time correlated with four AH symptoms including overall AH severity. Total AH severity accounted for a moderate amount of variance (11%) in the time to complete the TMT-B. These findings confirm that alcohol consumption negatively affects sleep, which is related to higher next-day hangover severity ratings and poorer cognitive performance.

## 1. Introduction

Alcohol hangover (AH) is described as an array of negative physical and mental symptoms that develop after a single episode of heavy alcohol consumption when the blood alcohol concentration (BAC) is nearing 0.00% [1,2]. AH typically follows alcohol consumed to an equivalent BAC of ≥0.11%, although recent research indicates that it can also occur after relatively low doses [3,4]. Common AH symptoms include headache, thirst and nausea, as well as sleep-related complaints such as fatigue and drowsiness which are often rated as the most severe [5,6,7]. Several studies have also found AH-induced impairments in cognitive functions including attention, memory, psychomotor speed and executive functioning [8,9,10,11,12,13]. Impaired cognitive functioning may be a factor in AH-related workplace absenteeism and reduced productivity, which costs the UK an estimated GBP 1.4 billion [14] and the US over USD 249 billion annually [15].

Many physiological mechanisms contribute to the presence of AH symptoms. These include, but are not limited to, dehydration, hormonal imbalances, oxidative stress and inflammation [16,17,18]. Biological factors such as age, as well as beverage congeners, which are toxic substances in alcoholic beverages other than ethanol (e.g., methanol), can also contribute to the severity of AH symptoms [10,19,20]. Another potential contributor to AH severity is the effects of alcohol intoxication on sleep architecture. Consuming alcohol prior to bed can have a negative influence on sleep by altering biological sleep rhythms [21,22]. Specifically, at high concentrations, alcohol has sedative properties and is often still being metabolised at sleep onset [10,21]. The body adjusts to this by reducing sleep onset latency, that is, the time it takes to fall asleep, as well as delaying rapid eye movement (REM) sleep and increasing the time spent in deep slow-wave sleep [10,21,23]. However, in the second half of sleep when blood alcohol concentrations decline, compensatory changes known as “rebound effects” occur [21]. This consequently increases REM and wake periods (i.e., the number and/or duration of awakenings after sleep onset), ultimately reducing sleep quantity and quality.

Despite this understanding, relatively few studies have explored the relationship between AH and sleep. Some existing research comes from studies using an experimental research design. Experimental designs involve inducing an AH state in a laboratory setting and controlling alcohol administration, as well as food and water intake and the time allocated to sleep [24]. One experimental study used polysomnography to monitor sleep following alcohol administered to a breath alcohol concentration (BrAC) of 0.11% [10]. Findings revealed an increase in wake periods (duration of awakenings) and a decrease in sleep efficiency (i.e., the proportion of time in bed that was actually spent sleeping), characteristic of high-dose consumption. Poorer self-reported sleep quality was also found, but there was no difference in total sleep time (TST). In contrast, another experimental study found no difference in sleep quality following alcohol administered to a peak mean BrAC of 0.155% [12]. A third study where alcohol was administered to a mean BrAC of 0.115% found no reduction in TST, but even improved sleep quality following alcohol compared with placebo [25]. Common to these conflicting studies was a short alcohol administration period (30 min–1.5 h) and 7–8 h of allocated sleep. Other limitations of experimental studies include ethical restrictions on the dose (generally capped at 1 g of alcohol per kilo body weight) and type of alcohol administered, which may also contribute to mixed outcomes [8,24].

Other AH studies have used semi-naturalistic research designs to study the association between AH and sleep. Unlike experimental designs, semi-naturalistic designs allow participants to self-initiate drinking in a familiar environment and attend a laboratory for testing the following morning when experiencing AH (for a further review, see Verster et al. [24]). Semi-naturalistic designs have high ecological validity and demonstrate that drinking frequently occurs at the expense of sleep, intensifying AH severity [24,26,27]. Verster et al. [7] found that after alcohol, time to bed was delayed by an average of 2 h, TST decreased by 1.5 h and the reduced TST negatively correlated with “being tired”. Self-reported sleep quality was also poorer after alcohol, a finding consistent with other semi-naturalistic studies [26,28], some of which additionally found reduced sleep onset latency [29,30] and increased awakenings (number of awakenings) after sleep onset [4]. However, in one study, TST did not decrease following alcohol when this was assessed via the levels of movement recorded on an accelerometer (wearable technology), as well as self-report measures [28].

While most of these studies have reported at least some association between AH and poor sleep, research design and other methodological differences such as the sleep variables assessed have resulted in inconsistencies [8,31]. This indicates research is needed to further elucidate this relationship. This could be approached in two ways. Firstly, an alternative naturalistic research design could be used. In a naturalistic design, participants are recruited when already out drinking, with on-field collected BrAC readings [32]. AH and sleep are then conveniently assessed at home using online measures. This research design has several advantages including the ability to obtain BrAC readings without influencing any prior drinking behaviours. In addition, sleep characteristics are not disrupted by the requirement to attend a testing site at a specified time, which is a common factor of both experimental and semi-naturalistic designs. This could provide more insight into normal sleep patterns following alcohol.

The second approach to further understand AH and sleep is to examine the severity of specific AH symptoms and compare these with the variety of sleep variables assessed in previous research. This includes the number and duration of nightly awakenings that have only been considered in two of the studies mentioned previously [4,10]. Two recent survey studies somewhat conducted this by comparing individual AH symptoms and select sleep measures [33,34]. In one study, headache, weakness, dizziness, hot/cold flushes and stomach pain correlated with TST [33]. The second study reported similar correlations using sleep quality and daytime sleepiness [34]. However, in both studies, limited sleep characteristics were assessed and AH was only measured retrospectively, which may partially explain the mostly weak correlations (i.e., *r* < 0.2).

A third factor that very few studies have examined is whether sleep is a compounding influence on the relationship between AH severity and cognitive performance [10]. Previous studies assessing the within-subject effects of AH have noted impairments in selective and sustained attention [13,35], psychomotor speed [11,36,37] and memory recall [36,38,39]. Rohsenow et al. [10] also found that AH severity positively correlated with reaction times on two tasks of sustained attention (i.e., the psychomotor vigilance task, and a continuous performance test). However, although higher AH severity also resulted in a lower sleep efficiency and REM percentage, these measures did not mediate the relationship between AH severity and task performance. This is somewhat unusual considering disrupted sleep has been observed to negatively impact cognitive performance across similar domains to AH. A meta-analysis on the cognitive effects of sleep deprivation indicated that the speed and accuracy of performance on simple and complex attention, working memory and information processing were all impaired following short-term sleep disruption (<48 h) [40]. In some cases, partial sleep loss (<5 h sleep) has shown to be more detrimental to cognitive functioning than short-term (<48 h) or long-term (>48 h) sleep deprivation [41].

The Trail-Making Test B (TMT-B) is a validated and reliable measure of cognitive flexibility, attention and working memory [42,43]. Previous research has shown the TMT-B to be sensitive in determining impairments relating to sleep loss [44,45,46] as well as the effects of acute alcohol [47,48,49]. Recent AH studies exploring higher-order functions including cognitive flexibility [39], working memory updating [9,50] and reward learning [51] have also reported impairments in the speed and/or accuracy of performance during AH compared to non-AH conditions. Therefore, it is worth considering whether these impairments are also related to the severity of AH and whether this is exacerbated by poor sleep.

The aim of the current study was to adopt a naturalistic study design to further explore the relationship between AH symptom severity and quantitative and qualitative aspects of sleep using multiple sleep characteristics. Based on previous research, the variables of interest in relation to sleep include the time to bed, time to fall asleep (i.e., sleep onset latency), the number and duration of awakenings after sleep onset, the total sleep time and sleep quality. A further aim was to determine whether sleep characteristics found to be strongly related to AH act as a mediating influence over the relationship between AH severity and performance on the TMT-B.

## 2. Materials and Methods

### 2.1. Design

This study formed part of a larger series of projects aimed at investigating the drinking patterns and motivations for drinking in an Australian sample [52,53]. The larger ‘Last Drinks’ study [53,54] included breathalysing and collecting data pertaining to alcohol consumption from 2516 individuals over several months. The current study involved a subset of the larger cohort (*n* = 346). Data for this study was collected during one month of the larger study. This study was a naturalistic design whereby participants were recruited after an evening of drinking as they were leaving entertainment districts of an Australian state capital (Brisbane) and were then contacted the following morning to complete online measures. This study was approved by Griffith University (2015/704) and Swinburne University Human Research Ethics Committees (SUHREC, 2016/167).

### 2.2. Participants

Participants were recruited at exit points around the central night-time entertainment districts of Brisbane. To be eligible, participants were required to be on their way home after an evening of drinking, be 18 years of age or over and proficient in the English language. Participants were also required to have a mobile phone and access to a desktop, laptop or tablet computer to complete next-day assessments.

### 2.3. Measures

#### 2.3.1. Breath Alcohol Content (BrAC)

Alcohol content was measured via expired breath using an Alcolizer LE5 (Alcolizer Pty Ltd., Perth, Australia). This device was calibrated by Queensland police, is Australian standard 3547 certified and has an accuracy of >0.005 at 0.100 BrAC g/100 mL [34]. Field research measuring intoxication levels in people attending night-time entertainment districts found this device to be reliable and valid [55].

#### 2.3.2. Alcohol Consumption

Participants were asked to state the number of standard drinks (i.e., 1 unit = 10 g pure alcohol in Australia) consumed the previous evening and hours spent drinking [56]. Drink options included beer/cider, wine, spirits (alone), spirits with mixed beverages and alcohol mixed with energy drinks. Information on the specific spirits consumed to ascertain the level of beverage congeners consumed was not collected.

#### 2.3.3. Alcohol Hangover Severity Scale (AHSS)

The AHSS assesses the severity of AH symptoms on an 11-point Likert-type scale ranging from 1 (absent) to 11 (extreme) [57]. Individual scores are added and averaged to indicate total AH severity. An 11-item version of the scale was used with the original item “concentration problems” being omitted. This was to reduce expectancy effects associated with engaging in a task requiring focussed attention [32]. The AHSS has been found to have good reliability and validity (Cronbach’s α = 0.85) [57].

#### 2.3.4. Core Consensus Sleep Diary (CSD)

The CSD is a self-report measure used for summarising quantitative and qualitative aspects of sleep [58]. The CSD was composed of 7 items relating to sleep quantity and a single eighth item relating to sleep quality. For sleep quantity, participants responded to questions such as “what time did you go to bed” and “what was the time of your final wake up” in 12 h time to the nearest 5 min interval (e.g., 8:00 a.m., 8:05 a.m., 8:10 a.m.). Sleep quantity items were “time to bed”, “sleep onset latency”, “number of awakenings”, “duration or nightly awakenings” and “time of final waking”. Responses to these 5 items were also calculated to determine the total sleep time and total time in bed, creating seven sleep quantity scores.

For the single item for sleep quality, participants were asked to indicate “how would you rate the quality of your sleep last night” on a 5-point Likert-type scale ranging from 1 (very poor) to 5 (very good). In addition, to assess whether sleep quality differed from a normal night’s sleep, participants were also asked to rate their sleep quality for a normal night’s sleep (i.e., “on nights when you have not been drinking alcohol, how would you rate the general quality of your sleep”), which was measured on the same 5-point scale.

#### 2.3.5. Online Trail-Making Task (TMT-B)

The TMT-B assesses cognitive flexibility, attentional set shifting and working memory [43]. The task includes letters (A–L) and numbers (1–13) presented in boxes in a random 5 × 5 grid pattern. Participants had to click on the boxes in an alternating digit/letter pattern (i.e., 1-A-2-B-3-C) as quickly and accurately as possible. A red flash in the box indicated an incorrect response. Outcome measures were completion time in seconds and total errors. Previous research found a high correspondence between the traditional pencil-and-paper and the online version [32].

### 2.4. Procedure

Researchers were stationed at major public transport hubs including taxi ranks and train stations in two locations across Brisbane, Australia. As part of the larger research project, participants were randomly approached on their way out of the bar districts between 12 a.m. and 5 a.m. and were presented with a verbal summary of the nature of the whole project and the specific study requirements. Every fourth person was initially asked to participate, and, after a refusal, every next person (or group) was approached (see Devilly, et al. [53]. Informed consent was obtained by the participants’ verbal agreement to be interviewed and to participate in night-time breathalyser measure assessments (as approved by both ethics committees). Participants willing to be interviewed were given a unique identifier card that had a link to the study information sheet (www.last-drinks.com) if they wished to obtain further information or to anonymously withdraw their data. Next, participants provided demographic information (i.e., age and gender) and completed a survey on their consumption that evening (results reported in Devilly et al. [53]). A BrAC reading was then taken, and participants were not restricted from knowing their result. Any participant with a BrAC reading ≥0.05% was invited to participate in the current study and complete online measures the following morning. The risk of bias from expectancy effects the following morning was thought to be minimal as previous research found no difference in cognitive performance during AH when participants were aware or not of the study purpose [59].

Consenting participants provided their mobile contact details and an estimated bedtime. Participants were contacted 8 h after their estimated bedtime via personal text message as a reminder to complete the online measures (i.e., 8 h represents an average night’s sleep and was the allowance used in previous experimental studies [10,25]). The text message contained a unique identifying number and directions to the secure website. Participants completed the online measures in a private residence in the following order: resupply of demographic information and alcohol consumption, AHSS, CSD and TMT-B. Alcohol consumption was recollected to account for any further alcohol that may have been consumed after leaving the entertainment districts. Total participation time did not exceed 15 min. At completion, participants were directed to another website to be compensated with an AUD 15 voucher.

### 2.5. Analysis

Data were analysed using SPSS version 27 (IBM Corp, Armonk, NY, USA). Initial screening revealed eight cases missing >30% of their total data. These cases were removed. A further nine participants had missing or spurious data on a single sleep variable. In order to calculate total sleep time and total time in bed, individual missing data points were replaced using a surrogate variable (e.g., “time out of bed” was used to replace “time of final wake up” and vice versa) [60]. One significant multivariate outlier was detected and removed. Initial inspections of histograms and P–P plots indicated most variables were skewed with the exception of total AH severity and select sleep variables (i.e., time of final wake up, total sleep time and total time in bed) which assumed normality. To correct for skewness in TMT-B completion times (s), log transformations were applied to the data and all subsequent analyses were run using both corrected and uncorrected scores. In other cases, transformations were unable to correct for violations of normality due to a high proportion of scores being close to or equalling zero (e.g., sleep onset latency, the number of nightly awakenings, duration of nightly awakenings and TMT-B errors) or a high number of extreme scores (e.g., 12% of scores for “fatigue” were rated as extremely high). However, examination of scatterplots indicated that all AH, sleep and performance variables formed reasonably monotonic relationships with all other AH, sleep and performance variables. Therefore, non-parametric statistics were employed.

Data analysis occurred in two phases. In the first phase, to confirm whether sleep quality was poorer after a night of drinking, the single item for sleep quality from the CSD was compared with the similar item measuring a normal night’s sleep using a two-tailed paired samples *t*-test. To account for any issues with non-normality, non-parametric Wilcoxon signed-rank tests were employed to confirm or reject the null. Next, the relationships between AH symptoms, sleep characteristics and TMT-B performance were analysed using two methods. Variables that assumed normality (i.e., total AH severity, time of final wake up, total sleep time, total time in bed and TMT-B completion times, which was corrected to normal) were compared using Pearson’s correlations. All other variables were assessed using non-parametric Spearman’s rank correlations. In the second phase, as determined through the results of Pearson’s correlations only (due to any potential issues with non-linear relationships), sleep variables that had formed a relationship with both AH severity and TMT-B performance (that is, only total sleep time) were subject to a Preacher and Hayes mediation analysis [61]. As age is also considered a possible influence on AH and sleep, the mediation analysis was also run with age as a potential covariate.

## 3. Results

### 3.1. Participant Characteristics

Of the 346 participants who consented to the current study, 108 completed next-day measures (31% participation rate) of which 99 provided usable data sets. As outlined in Scholey, et al. [32], there was no difference in the level of intoxication between those who did and did not participate in next-day measures.

Participants were 52% male and had a mean age of 24.16 (±5.68; range 18–49) years. On the evening of drinking, participants consumed an average of 13.85 (±6.38) drinks over 7.55 (±3) h, resulting in a mean BrAC of 0.111% (±0.04) at the time of leaving the entertainment districts. Table 1 displays the means and standard deviations for AH symptoms, sleep characteristics and TMT-B performance. To ease interpretation, uncorrected scores for TMT-B (s) are presented. As shown in Table 1, participants went to bed after 3 a.m. on average, with the TST just less than 5 h. For AH symptoms, “fatigue” had the highest mean severity, followed by “thirst”. All other symptoms including total AH severity were rated as mild (<3) to moderate (3–6) in severity.

### 3.2. Sleep Quality

To confirm whether sleep quality following an evening of drinking differed from a normal night’s sleep, the single sleep quality item from the CSD (i.e., “how would you rate the quality of your sleep”) was compared with the similar single sleep quality item measuring a normal night’s sleep. Sleep quality after an evening of drinking was found to be significantly poorer, *t*(98) = 2.90, *p* = < 0.01, *d* = 0.4, CI [0.13, 0.7], with a moderate effect size.

### 3.3. Correlations

Table 2 presents the correlations for drinking characteristics, AH symptoms, sleep and task performance.

#### 3.3.1. Drinking Characteristics

As shown in Table 2, hours drinking and the total drinks consumed correlated with the time to bed as well as completion times on the TMT-B. Hours drinking also correlated with total sleep time and the total time in bed. BrAC did not correlate with any sleep or performance variables (for correlations between drinking characteristics and AH severity, see Scholey et al. [32]).

#### 3.3.2. AH Severity and Sleep Characteristics

As shown in Table 2, total AH severity was related to the most sleep characteristics, positively correlating with the number and duration of nightly awakenings and negatively correlating with total sleep time and sleep quality. The single sleep characteristic that correlated with the most other AH variables was the duration of nightly awakenings, which correlated with six individual AH symptoms. The number of nightly awakenings, total sleep time and sleep quality also correlated with three individual AH symptoms, of which the latter two formed moderate negative correlations with the single item “fatigue”. The time of final wake up and total time in bed only correlated with one individual AH item, while the time to bed and sleep onset latency did not correlate with any AH symptoms.

#### 3.3.3. AH Severity, Sleep and TMT-B Performance

Times to complete the TMT-B (s) positively correlated with total AH severity as well as seven other individual AH symptoms. TMT-B completion times also negatively correlated most with total sleep time as well as total time in bed, and positively correlated with time to bed. The single item “fatigue”, time of final wake up and total time in bed were the only variables related to the number of errors on the TMT-B.

### 3.4. Mediation

**Figure 1:**
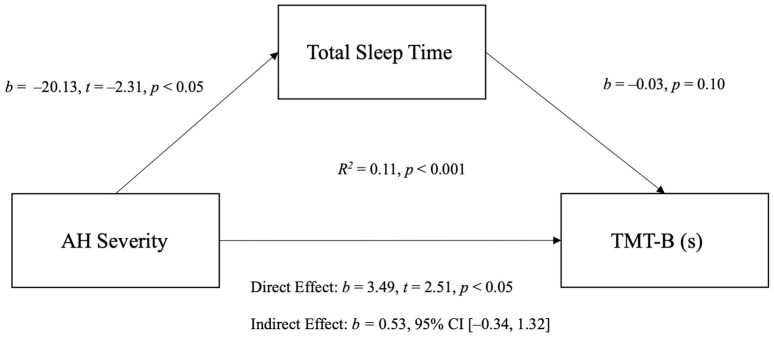
Mediation model for the relationship between AH severity, TMT-B (secs) and total sleep time.

Although “fatigue”, “thirst” and “stomach pain” correlated with both TMT-B (s) and TST, these correlations were conducted using non-parametric statistics and as such were not appropriate for any further mediation analysis. Given that TST was the only sleep variable that correlated with both total AH severity and TMT-B (s), a mediation analysis was conducted with TST as the mediating variable between total AH severity and TMT-B (s). Analysis of log-transformed and uncorrected scores on the TMT-B (s) revealed similar outcomes, so for ease of interpretation, the untransformed scores are presented. As shown in Figure 1, the findings reveal a significant overall model for predicting completion times on the TMT-B from AH severity and TST (*F*(1.97) = 5.81, *p* < 0.01), with 11% of the variance in completion times accounted for by the model. However, TST did not mediate the relationship between AH severity and cognitive performance. Further mediation analyses using age as a covariate revealed no significant differences to outcomes (see Appendix A).

## 4. Discussion

The current study explored the relationship between AH severity, sleep characteristics and cognitive performance using a naturalistic study design. Participants were recruited on their way home from an evening of drinking and completed AH and sleep measures as well as the TMT-B. The findings reveal that six out of the eight sleep characteristics correlated with at least one AH symptom. Duration of nightly awakenings correlated with the most AH symptoms. Total AH severity correlated with four sleep measures as well as TMT-B completion times. Several AH symptoms, as well as total sleep time (TST), also correlated with completion times on the TMT-B (s). However, TST did not mediate the relationship between AH severity and TMT-B (s) performance.

Firstly, our findings confirm that a night of drinking resulted in poorer subjective sleep quality compared with a normal night’s sleep. This is in line with the majority of previous studies [4,7,10,29], but not all [12,25]. In relation to individual AH symptoms, the duration of nightly awakenings correlated with the most AH symptoms, that is, six individual symptoms (clumsiness, thirst, sweating, shivering, nausea and heart pounding) and total AH severity. The number of awakenings also correlated with three individual symptoms (sweating, shivering and heart pounding) and total AH severity. Nightly awakenings are not commonly assessed in AH research, although Rohsenow et al. [10] similarly found that a longer duration of nightly awakenings resulted in higher AH severity using polysomnography. Likewise, a semi-naturalistic study found an increase in the number of nightly awakenings after consuming alcohol [4]. These findings highlight that experiencing nightly awakenings has a significant influence on AH symptom severity and should be included in future research.

Other sleep characteristics that correlated with multiple AH symptoms were TST and sleep quality. Less TST and poorer sleep quality commonly resulted in higher ratings of fatigue and overall AH severity. These findings are similar to previous correlational research [7,33,34] and somewhat reflect other semi-naturalistic studies that found a within-subject reduction in TST [29] and/or poorer sleep quality during AH compared with non-AH conditions [28]. However, they differ from some experimental studies where non-significant differences in TST have been observed [10,25]. This could be due to the influence of experimental research on sleep hygiene. As such, in experimental research, bedtimes are generally scheduled and significantly earlier (before 12 a.m.) than those found in the current study (after 3 a.m.). The time allocated to sleep in these experimental studies is also adequate (7–8 h), sometimes with distractions, such as mobile phones, not allowed [10]. These factors promote good sleep hygiene and could unintentionally improve TST regardless of alcohol consumed. On the other hand, some of these studies had the advantage of measuring sleep objectively rather than relying on self-reported sleep. Considering self-report measures are prone to recall bias, this could alternatively explain the conflicting findings [28].

The findings for TMT-B performance reveal completion times to be related to seven individual AH symptoms, total AH severity and three characteristics of sleep. Total AH severity also accounted for a moderate but significant amount of variance in TMT-B completion times. Together, these findings were not unexpected given recent AH studies have reported within-subject effects of AH on various executive functions [9,39], with sleep disruptions also showing a similar relationship to cognitive impairment [40]. Despite this, when TST was included as a potential mediating variable, no mediation was observed in a model with overall AH severity. This provided no evidence that the association between AH severity and cognitive performance is mediated by sleep disturbances. This is similar to a previous study even though different sleep variables (sleep efficiency and REM %) and cognitive measures were used (i.e., the psychomotor vigilance task and a continuous performance test) [10].

The reasons for a lack of mediation are not clear. Considering several other AH variables including “fatigue” also correlated with TMT-B (s), and these variables make up the average AH score, there is already some overlap in these constructs [10]. In addition, as various physiological mechanisms contribute to the range of AH symptoms, it may be the combination of these complex connections to AH impairment that has a greater impact over sleep loss alone. As an example, dehydration is a mechanism of AH that is related to poor concentration due to alcohol-induced changes in the anti-diuretic hormone vasopressin [16,17,62]. An alcohol-related immune response has also been associated with impaired memory performance due to the actions of increased concentrations of pro-inflammatory cytokines [18,63]. This, combined with poor sleep, may be one of the factors that contributes to poorer cognitive performance and that as assessed by the TMT-B.

The remaining sleep variables had little to no relationship with drinking, AH and cognitive performance. Although time to bed, time of final wake up and total time in bed correlated with few AH symptoms and/or TMT-B errors, these correlations were no higher than *r_s_* = 0.21 (except for time in bed and “fatigue”, where *r_s_* = 0.24), meaning the relevance of these findings is questionable. Drinking characteristics also correlated with few variables. The hours of drinking and total amount of drinks consumed delayed time to bed, suggesting that a night out drinking occurs at the expense of sleep [26]. Similarly, the hours of drinking also reduced the total sleep time and total time in bed. However, BrAC was not related to any measure. This might be considered unusual as higher concentrations of alcohol can have a more pronounced effect on sleep architecture [21]. However, BrAC was also only taken when participants were on their way home and may not reflect the peak BrAC obtained. Other research has also suggested that subjective intoxication is a more important contributor to AH severity over BrAC, so this may also explain some of the lack of findings here [64].

Regarding other findings, the single item “fatigue” was the only AH symptom related to TMT-B errors. It is difficult to draw any strong conclusions here considering 53% of the sample made no errors on the TMT-B and a further 25% made only one or two errors [34]. We have previously suggested that the impairment in speed on the TMT-B and relatively few errors may signify a differing effect of AH on the speed–accuracy trade-off (SATO) compared with that of acute intoxication [32]. This is due to several acute intoxication studies finding a characteristic shift in SATO with higher errors compared to relatively limited effects on response speed [65,66,67].

The findings in this study support growing evidence that AH is associated with poor sleep and cognitive performance. Adopting a naturalistic study design allowed for a unique perspective on drinking, AH and sleep. Although the mean scores on individual AH items were mostly low to moderate in severity (barring the single-items ‘fatigue’ and ‘thirst’), these scores are highly consistent with previous research [68], including studies that reported a significant difference in symptom severity from control conditions and other correlational research assessing AH and sleep [33,34]. The BrAC readings obtained also reflect previous research, which suggests that sample selection was not biased and was representative of end-of-night intoxication [53,54]. However, a significantly later time to bed and lower TST were found compared with other studies. This could be considered a study strength, as it highlights the potential limitations of other research designs to obtain ecologically valid sleeping behavior after drinking. Another study strength was the cross-section of ages recruited. As previous research typically focussed on younger age groups, the current research emphasises that AH is not just experienced by younger drinkers. In addition, including age as a potential covariate in the mediation model did not change the outcomes.

This study was not without limitations. Firstly, participants were not screened for dependency issues, concurrent use of other psychoactive substances and sensitivity to AH. These factors have been causes for exclusion in previous research and have the potential to bias the results obtained in this study [26,69]. Secondly, although participants were on their way home for the evening, BrAC may not be reflective of the peak BrAC obtained, especially if participants consumed more alcohol once at home or had only recently finished drinking. Moreover, BrAC the following morning was not assessed, meaning the presence of AH (classified as <0.02% BrAC [8]) could not be confirmed.

Thirdly, this study was conducted in only one major location, which may limit generalisability to other cultures and societies. Limited generalisability could also be extended to the use of only one short cognitive task. Whilst the TMT-B has been shown to be a valid and reliable measure of executive functioning, it is difficult to generalise the negative effect of AH severity observed here to impairments in cognitive performance more broadly [42].

Further limitations of this research relate to overall AH severity. Recent research has indicated that a single-factor overall AH severity score may be a more reliable indicator of true AH severity over the average of individual AH items [70]. This is due to AH questionnaires not representing all possible AH symptoms, as well as variability in the presence and severity of common versus less frequent AH symptoms in the scale. As suggested previously, there was already potential overlap in the mediation model using the averaged overall AH severity, which includes the single-item score for “fatigue”. Therefore, including a single-factor score may be more appropriate for any future research considering mediation.

Consistent with past AH research, the reliance on self-report measures which can be prone to bias is another limitation of the current study. This increased risk of bias also includes not restricting participants from knowing their BrAC results (i.e., potentially increasing the risk of expectancy effects the next morning) and asking participants about a normal night’s sleep on the morning when experiencing AH. The use of objective sleep measures such as those used by Devenney et al. [28] was not possible for this type of research design but would add valuable insight into research on AH and sleep in the future, as would the use of experimental and within-subject research designs. As previous studies have also highlighted sex differences in sleep characteristics following alcohol [12,33,71], and an influence of beverage congeners on AH severity, this may be considered a possible limitation that could also be explored in future research.

## 5. Conclusions

Using a naturalistic study design, this study confirmed a relationship between AH symptom severity and several sleep characteristics. An exploration of the correlational relationship revealed the duration of nightly awakenings as the sleep variable that related to the most AH symptoms. This suggests waking after sleep onset should be consistently included in future AH research. AH severity also accounted for variance in a task of executive functioning, which has potential implications for complex task performance (such as driving) with an AH. However, TST was not a mediating influence on the relationship between AH severity and performance. Given this lack of mediation, future research could explore how other mechanistic variables such cytokine concentrations and dehydration may contribute alongside sleep to the complex relationship between AH severity and cognitive functioning. Future research should also consider the use of advanced technologies to obtain objective measures of sleep as well as within-subject methodologies to strengthen the above findings. 

## Figures and Tables

**Table 1:** Means and standard deviations for AH and sleep characteristics.

Variable	Mean	SD
Sleep Characteristics		
Time to bed (h:m)	3:41	1:07
Sleep onset latency (h:m)	0:32	0:47
No. nightly awakenings	1.32	1.84
Duration of nightly awakenings (min)	0:07	0:16
Time of final wake up (h:m)	9:29	1:48
Total sleep time (h:m)	4:58	2:06
Total time in bed (h:m)	6:32	1:50
Sleep quality	3.31	1.15
Normal sleep quality	3.73	0.9
AH Symptoms		
Total AH severity	3.85	1.42
Fatigue	7.55	2.49
Apathy	4.57	2.88
Clumsiness	3.86	2.51
Confusion	2.76	2.19
Thirst	7.15	2.47
Sweating	3.08	2.53
Shivering	1.25	1.05
Stomach pain	2.72	2.56
Nausea	3.87	2.78
Dizziness	3	2.27
Heart pounding	2.55	2.36
Cognitive Performance—TMT-B		
Completion time (s)	55.29	20.12
Number of errors	2.57	7.19

**Table 2:** Correlation coefficients for sleep characteristics, drinking characteristics, AH symptoms and cognitive performance.

Variables	Sleep Characteristics								Cognitive Performance	
	Time to Bed	Sleep Onset Latency	N°. Nightly Awakenings	Duration of Nightly Awakenings	Time of Final Wake Up	Total Sleep Time	Total Time in Bed	Sleep Quality	TMT-B (s)	TMT-B (Err)
Drinking Characteristics										
BrAC	0.160	−0.169	−0.010	−0.035	0.096	0.041	−0.152	0.040	0.186	−0.028
Hours drinking	0.341 **	−0.039	0.197	0.182	−0.033	−0.238 *	−0.258 **	−0.164	0.294 **	0.108
Total drinks	0.235 *	0.037	−0.018	−0.033	0.073	−0.076	−0.165	0.025	0.212 *	0.102
AH Symptoms										
Total AH severity	0.174	0.024	0.252 *	0.302 *	0.032	−0.229 ^a^	−0.112	−0.236 *	0.286 ^b^	0.114
Fatigue	0.180	0.085	0.153	0.188	−0.164	−0.320 **	−0.241 *	−0.309 **	0.289 **	0.219 *
Apathy	0.090	0.001	0.033	0.117	0.222 ^a^	0.028	0.093	−0.100	0.131	−0.067
Clumsiness	0.066	0.099	0.162	0.216 *	0.058	−0.193	−0.028	−0.153	0.199 *	−0.003
Confusion	0.178	0.028	0.117	0.162	0.027	−0.126	−0.167	−0.203 *	0.253 *	0.101
Thirst	−0.011	0.055	0.132	0.204 *	−0.171	−0.243 *	−0.165	−0.169	0.253 *	0.052
Sweating	0.119	0.030	0.251 *	0.218 *	−0.044	−0.116	−0.133	−0.182	0.161	0.102
Shivering	0.053	−0.075	0.341 *	0.280 **	0.014	−0.158	−0.021	−0.193	0.087	0.010
Stomach pain	0.196	0.059	0.163	0.138	−0.018	−0.263 *	−0.177	−0.184	0.203 *	0.138
Nausea	0.090	−0.028	0.145	0.202 *	0.024	−0.048	−0.037	−0.060	0.234 *	0.052
Dizziness	−0.053	−0.031	0.140	0.141	0.003	−0.080	0.099	−0.039	0.125	−0.019
Heart pounding	0.163	−0.018	0.308 **	0.276 **	0.041	−0.101	−0.031	−0.241 *	0.292 **	0.137
Cognitive Performance										
TMT-B (s)	0.214 *	−0.013	0.118	0.191	−0.188	−0.235 ^a^	−0.207 *	−0.130	-	-
TMT-B (Err)	0.148	−0.032	−0.080	−0.041	−0.201 *	−0.145	−0.210 *	−0.005	-	-

**Note:** Significant differences in Spearman’s rank correlations (*r_s_*) denoted as * *p* < 0.05, and ** *p* < 0.001; significant differences in Pearson’s correlations (*r*) denoted as ^a^ *p* < 0.05, and ^b^ *p* < 0.001.

**Figure A1:**
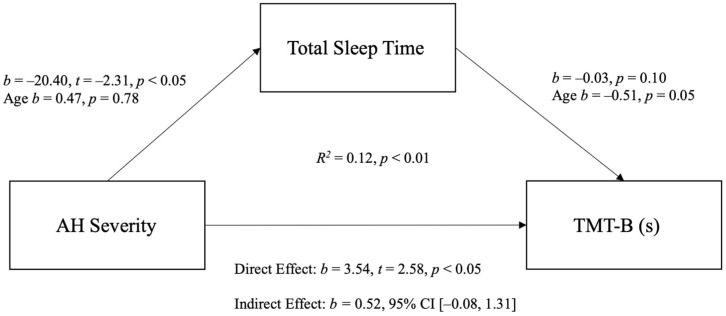
Mediation model for the relationship between AH severity, total sleep time and TMT-B completion times, with age as a covariate.

## Data Availability

The data presented in this study are available on request from the corresponding author.

## Appendix A

The relationship between AH severity, TMT-B (s) and total sleep time, with age as a covariate.

Observations of Mahalanobis’s distance revealed two outlying cases when considering age as a potential covariate in the mediation model. Removal of these outliers did not change the significance of any relationship, and thus these were retained in the analysis. Figure A1 presents the mediation model for the relationship between AH severity, TMT-B (s) and total sleep time with age as a covariate. The overall model was significant (*F*(1.96) = 6.42, *p* < 0.01), with 12% of the variance in TMT-B completion times accounted for by AH severity. With age as a covariate, the relationship between total sleep time and TMT-B (secs) was trending towards significance. However, total sleep time still did not mediate the relationship between AH severity and performance.
